# Too easy? The influence of task demands conveyed tacitly on prospective memory

**DOI:** 10.3389/fnhum.2015.00242

**Published:** 2015-04-30

**Authors:** Joana S. Lourenço, Johnathan H. Hill, Elizabeth A. Maylor

**Affiliations:** Department of Psychology, University of WarwickCoventry, UK

**Keywords:** prospective memory, attention allocation policy, task interference, tacit demands, metacognition, anticipated demands

## Abstract

Previous research suggests that when intentions are encoded, participants establish an attention allocation policy based on their metacognitive beliefs about how demanding it will be to fulfill the prospective memory (PM) task. We investigated whether tacit PM demands can influence judgments about the cognitive effort required for success, and, as a result, affect ongoing task interference and PM performance. Participants performed a lexical decision task in which a PM task of responding to animal words was embedded. PM demands were tacitly manipulated by presenting participants with either typical or atypical animal exemplars at both instructions and practice (low vs. high tacit demands, respectively). Crucially, objective PM task demands were the same for all participants as PM targets were always atypical animals. Tacit demands affected participants’ attention allocation policies such that task interference was greater for the high than low demands condition. Also, PM performance was reduced in the low relative to the high demands condition. Participants in the low demands condition who succeeded to the first target showed a subsequent increase in task interference, suggesting adjustment to the higher than expected demands. This study demonstrates that tacit information regarding the PM task can affect ongoing task processing as well as harm PM performance when actual demands are higher than expected. Furthermore, in line with the proposal that attention allocation is a dynamic and flexible process, we found evidence that PM task experience can trigger changes in ongoing task interference.

Prospective memory (PM) is ubiquitous in everyday life and even vital in some settings (e.g., remembering to remove surgical instruments from a patient before closing; Einstein and McDaniel, 2005). When forming an intention, what information do people use to decide how much effort they should expend to ensure that the intention will be successfully remembered? And how capable are people in dealing with unforeseen changes to planned intentions? To illustrate, one of the authors assumed that a pharmacy on the way home would be sufficient to cue remembering to fill in a prescription, but this failed because the shop no longer captured attention when its neon sign was not working.

Recent findings on the role of metacognition in PM have suggested that people’s estimates about their likelihood of successfully fulfilling intentions are generally well calibrated, although participants tend to be slightly underconfident about their performance (e.g., Knight et al., 2005; Meeks et al., 2007; Schnitzspahn et al., 2011). To determine the likelihood of success, participants rely on metacognitive beliefs about the cognitive demands of the entire task set (i.e., ongoing and PM activities) and their ability to perform the upcoming tasks (Marsh et al., 2005; Meeks et al., 2007; Einstein and McDaniel, 2008; Rummel et al., 2013). These evaluations can influence, for example, whether external reminders are used (Gilbert, 2015). They can also affect the attention allocation policy established by participants at the outset of the task, which specifies the relative weighting of attention to the ongoing and PM tasks (Marsh et al., 2003, 2005; Hicks et al., 2005; see also Smith, 2003; Einstein et al., 2005). The key question in the present study was whether available information about potential target events has any bearing on how attentional resources are devoted to the PM task.

Studies have shown an increase in task interference (i.e., slowing to the ongoing task) when PM task difficulty is increased by changing objective task demands such as number of targets or specificity of intentions (e.g., Hicks et al., 2005; Cohen et al., 2008; Lourenço et al., 2013). Also, when PM tasks are nonfocal (i.e., ongoing task processing does not direct attention toward processing the relevant features of the target), individuals devote extra resources to remembering the intention (e.g., Einstein et al., 2005; Scullin et al., 2010). Another approach has involved manipulating anticipated task demands through explicit instructions while leaving objective task demands intact. For instance, instructing participants that the PM task is more important than the ongoing task affects attention allocation as evidenced by an increase in both task interference and PM performance (e.g., Kliegel et al., 2004; Einstein et al., 2005). Similarly, Rummel and Meiser (2013; Experiment 2) showed that explicit information about the cognitive effort necessary for fulfilling a PM task influences attention-allocation strategies. Their manipulation of anticipated PM task demands by instructing participants that detection of the targets will be rather hard (vs. quite easy) significantly increased ongoing task costs.

Moreover, Boywitt and Rummel (2012; Experiment 1) manipulated anticipated task demands by instructing participants that targets would be presented for only 10% of all participants (or 90% in another condition). Using a diffusion model analysis, the authors showed that participants who expected the probability of target presentation to be low were less cautious in their responding (i.e., their response thresholds in the ongoing task were lower as revealed by the diffusion model’s response criterion parameter). Thus, participants” strategic approach to performing the ongoing task depended on anticipated PM task demands.

We addressed the question of whether *tacit* PM task demands can also affect participants’ effort and success in a nonfocal PM task. Rather than manipulating expected PM demands using explicit instructions—as in all prior work—we used a categorical (nonfocal) PM task and varied the particular target exemplars (typical vs. atypical) presented prior to the experimental trials. We predicted that when asked to give a PM response to animal words during an ongoing lexical decision task (LDT), participants instructed using typical exemplars of the target category (i.e., exemplars that are fluently processed and easily accessible in memory; Koriat et al., 2004) would expect to successfully accomplish the PM task with low effort and thus display smaller ongoing task costs than those instructed using atypical exemplars. Critically, objective task demands were kept constant such that all PM targets presented during the ongoing task were atypical animals. Hence, we also predicted that participants presented with typical exemplars at encoding would perform worse on the PM task because successful PM performance in nonfocal tasks requires the engagement of attention-demanding processes (e.g., Einstein et al., 2005).

Furthermore, we investigated whether the attention allocation policy set at the beginning is flexible such that it can be adjusted on the basis of experience with the PM targets or whether it is relatively immutable. Specifically, we examined whether incongruence between expected and actual PM task demands can lead to local changes in participants’ attention allocation policy. We predicted that participants given typical exemplars (low tacit PM demands) would adapt to the new demands and show increased task interference after realizing that targets could be atypical instances. This would provide evidence that individuals can use their experience with the PM task to adjust their strategies when expectations regarding the PM targets are biased.

## Method

### Design and Participants

The design was a 3 × 2 mixed factorial, with tacit PM demands (high, low, none) as the between-subjects factor, and block (baseline, PM) as the within-subjects factor. Participants were 90 undergraduate students (39 female) aged 18–23 years (*M* = 20.8, *SD* = 1.0). Thirty participants were randomly assigned to each of the three conditions. Testing took place individually in sessions lasting approximately 25 min. Ethical approval was granted by the Warwick Psychology Department’s Research Ethics Committee and all participants provided their informed consent.

### Materials and Procedure

Participants were first told about the LDT. Instructions stated that they had to decide as quickly and accurately as possible whether a string of letters was a word (“J” key press with right index finger) or not (“F” key press with left index finger). Following the opportunity to ask questions, participants performed 20 practice trials and then a baseline block (see Table 1) consisting of 10 buffer trials and 100 lexical decision trials (50 words and 50 nonwords).

**Table 1:** **Illustration of the main design and procedure for participants with high, low and none tacit prospective memory demands, with typicality of animals indicated in italics**.

	Tacit PM Demands		
	High	Low	None
Baseline Block	Lexical Decision Task (LDT)		
PM Instructions	Press “Y” to animal	Press “Y” to animal	—
	words (e.g., WALRUS)	words (e.g., DOG)
	*Atypical*	*Typical*
Practice (1 target)	LDT + PM task	LDT + PM task	LDT
	……raccoon……	……mouse……
	*Atypical*	*Typical*
Delay	Processing Speed Test + Questionnaire	
PM Block (4 targets)	LDT + PM task		LDT
	…….puffin…….gazelle…….boar…….hyena…….	
	*Atypical*	

Next, participants were told that they would perform a second block of the LDT, and additionally given the PM instructions. Those in the high and low tacit PM demands conditions were given the same PM task of responding to animal words, but were presented with different animal exemplars at both instructions and practice. These were atypical exemplars (*walrus* and *raccoon*) or typical exemplars (*dog* and *mouse*) in the high vs. low tacit PM demands conditions, respectively. Specifically, participants were instructed that if they ever saw an animal word (e.g., WALRUS or DOG, included in brackets according to condition) they should press the “Y” key after they made their lexical decision or as soon thereafter as they could. Participants explained the instructions to the experimenter (to confirm their understanding) before completing 20 practice trials, which included the presentation of an animal word (*raccoon* or *mouse*, according to condition) on Trial 15. To create a delay between PM task instructions and the start of the PM block, participants completed a 2-min test of processing speed and a demographic questionnaire. Those in the “none” PM demands condition went through the same procedure except that they did not receive the PM task instructions. The PM block comprised 10 buffer trials and 260 lexical decision trials, of which 256 were filler trials (128 words and 128 nonwords) and 4 were PM trials. PM targets (all atypical animals) were presented on Trials 101, 152, 203 and 254 (puffin, gazelle, boar, and hyena,^1^ respectively, for all participants).

Each trial consisted of a fixation cross presented for 250 ms, followed by the letter string in lowercase (30-pt font) until classified as a word/nonword, and finally a waiting message until the spacebar was pressed. Filler words in the LDT, matched with the PM targets on mean length, syllables, and frequency, were 4–7 letters, 1–3 syllables, and HAL frequency 5.5–7.5 according to Balota et al. (2007; nonwords with 4–7 letters were selected from the same source). At the end of the PM block, participants completed a questionnaire to test their recall of the intended action. Recall was perfect for all participants.

## Results

### Data Screening

Two participants in the high tacit PM demands condition who were more than 2.5 SDs from their group’s mean response time (RT) in the ongoing task were excluded. As is commonly observed in LDTs, performance was highly accurate with 93% of words identified correctly and no significant differences across conditions. Based on previous PM research (e.g., Knight et al., 2011), word RTs were trimmed to include only correct responses to words that were less than 2.5 SDs away from each participant’s mean. Trimming was done separately for the baseline and PM blocks (PM targets and the trial immediately following each of the targets were excluded) and resulted in the elimination of 2.6% of correct RTs.

### Ongoing Task Performance

**Figure 1:**
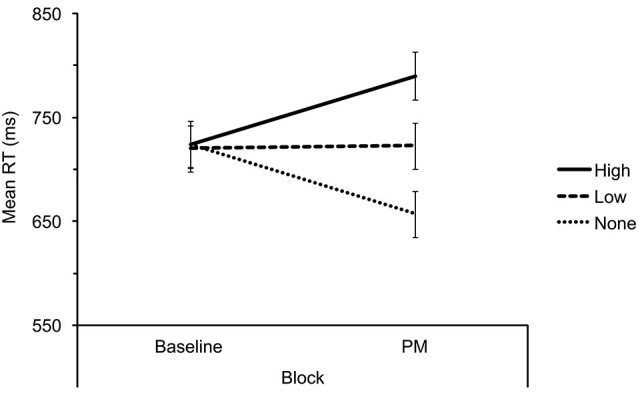
**Mean correct response time (RT) in milliseconds (ms) for lexical decisions to filler words for high, low and none tacit prospective memory (PM) demands conditions across blocks**. Error bars represent ± 1 standard error.

**Figure 2:**
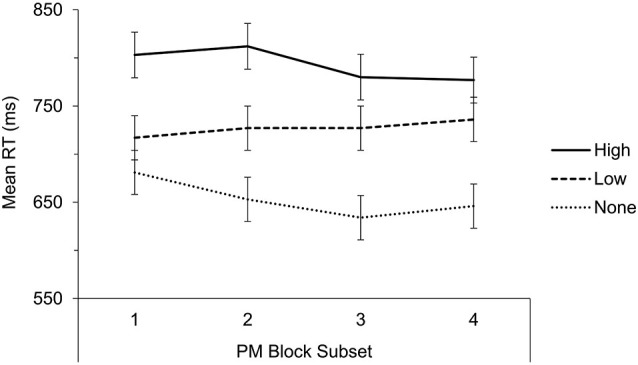
**Mean correct RT in milliseconds (ms) for lexical decisions to filler words across conditions and subsets in the PM block**. Error bars represent ± 1 standard error.

Our main question was whether tacit information about the PM targets at instructions/practice can influence expectations about PM task demands as reflected by task interference. Mean RTs on filler word trials were included in a 3 × 2 mixed ANOVA with tacit PM demands (high, low, none) as the between-subjects factor and block (baseline, PM) as the within-subjects factor (see Figure 1 for means). Neither main effect was significant (*p*s > 0.2) but the interaction was significant, *F*_(2,85)_ = 17.48, *MSE* = 3,731.21, *p* < 0.001, *η_p_*^2^ = 0.29. We therefore conducted two further mixed 2 × 2 (tacit PM demands × block) ANOVAs for high vs. low PM demands conditions, and low vs. none PM demands conditions, both yielding significant interactions (*p* = 0.006 and 0.003, respectively). We also divided the PM block into four subsets (i.e., correct word trials preceding each PM target; see Figure 2) and examined ongoing task cost for the first subset, namely, trials occurring before the first target presentation. The pattern of results was similar to that from the overall task interference analysis, with a significant interaction for the 3 × 2 ANOVA (*p* < 0.001), a significant interaction for the high vs. low demands ANOVA (*p* < 0.002), but this time only a marginally significant interaction for the low vs. none demands ANOVA (*p* = 0.074). Therefore, in line with our predictions, ongoing task cost was influenced by our manipulation of tacit PM task demands such that task interference in the low tacit demands condition was significantly lower than in the high demands condition and this was evident overall and before the first PM target occurrence.

While actual PM task demands did not differ between conditions, targets were consistent with expectations in the high but not in the low demands condition. Thus an important question is whether participants in the low demands condition adjusted their allocation of attention when PM task demands turned out higher than expected. To examine if task interference changed from the first to the fourth PM block subset, we included filler word RTs in a 3 × 4 mixed ANOVA with tacit PM demands (high, low, none) as the between-subjects factor and PM block subset (1–4) as the within-subjects factor (see Figure 2). There was a significant effect of tacit PM demands, *F*_(2,85)_ = 7.89, *MSE* = 71,628.20, *p* < 0.001, *η_p_*^2^ = 0.157, but no effect of PM block subset, *F*_(3,255)_ = 1.90, *p* = 0.13, and no interaction, *F*_(6,255)_ = 1.72, *p* = 0.117. Thus, RTs remained relatively stable throughout the PM block in all conditions, suggesting that participants in the low tacit demands condition allocated fewer resources to the PM task and also failed to adapt to the higher than expected attentional demands posed by the task (although it can be seen from Figure 2 that the trend toward an interaction reflects the resemblance of the low PM demands condition to the none PM demands condition in the first PM block subset but to the high PM demands condition by the fourth PM block subset).

However, of particular interest here is examination of task interference according to success to the first target presentation in the low tacit demands condition. Did participants who successfully detected the first target (*n* = 12) show subsequently increased task interference in comparison to those who failed (*n* = 18)? We included RTs in the low tacit demands condition in a 2 × 2 mixed ANOVA with first target (success, failure) as the between-subjects factor and PM block subset (1 vs. 2) as the within-subjects factor. Results revealed a significant interaction, *F*_(1,28)_ = 8.60, *MSE* = 64,211.91, *p* = 0.007, *η_p_*^2^ = 0.24, such that there was slowing from trials preceding to those succeeding the first target when target detection was a success (*M*s = 711 and 765 ms, *SDs* = 93 and 121, respectively; *t*_(11)_ = −2.84, *p* = 0.016), but not when it was a failure (*M*s = 722 and 701 ms, *SDs* = 120 and 112, respectively; *t*_(17)_ = 1.25, *p* = 0.230).^2^

### PM Task Performance

**Figure 3:**
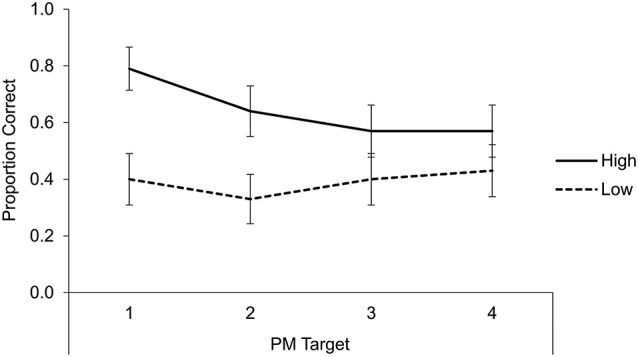
**Mean proportion correct for the PM task across conditions for each of the four PM targets**. Error bars represent ± 1 standard error.

Having shown that manipulation of tacit PM demands affected attention allocation policies, we next consider whether it also affected PM task performance. PM responses were scored as correct if participants pressed the “Y” key during the target trial or within the next trial (see Figure 3 for means). A 2 × 4 mixed ANOVA with tacit PM demands (high, low) and PM target (1–4) as between- and within-subjects factors revealed an effect of tacit PM demands, *F*_(1,56)_ = 8.34, *MSE* = 0.44, *p* = 0.006, *η_p_*^2^ = 0.13, such that PM performance was significantly better with high than with low tacit demands (0.64 vs. 0.39) with no other significant effects (both *p*s > 0.3).

## Discussion

Our data show that tacit PM task demands can affect participants’ effort and success in a PM task. Specifically, those given typical exemplars at encoding (low tacit demands condition) showed less task interference and worse PM performance than those given atypical ones, demonstrating that indirectly conveyed information about PM task difficulty (i.e., examples of targets at encoding) affects expectations about PM demands and thereby the amount of attention allocated to the intention. Crucially, our results also demonstrate that, although biased expectations can harm PM performance when actual demands turn out higher than expected, participants can adapt following target experience. In other words, the present results show that individuals can adjust attention-allocation strategies following successful detection of a target that is incongruent with their (biased) metacognitive expectations.

The present study provides novel evidence that detecting PM targets that are inconsistent with tacit demands can elicit local changes in attention allocation. Specifically, participants in the low tacit demands condition who detected the first PM target (hence realizing that targets could be atypical exemplars) showed an increase in ongoing task RTs following this first target and also went on to perform similarly to those in the high tacit demands condition (0.67 vs. 0.64 success to Target 2). This extends previous research showing that individuals can adjust the amount of attention devoted to the intention when monitoring goes unreinforced due to the lack of PM target occurrences (e.g., Loft et al., 2008; Scullin et al., 2010; cf. Boywitt and Rummel, 2012), and also that trial-by-trial changes in the allocation of attention can occur. For example, task interference can change flexibly as a result of changes in the effort toward an ongoing task (Marsh et al., 2005) or an item’s relevance for the PM task (e.g., Marsh et al., 2006; Lourenço and Maylor, 2014). The present results provide support for the proposal that attention allocation is flexible (Scullin et al., 2013), such that experience with the ongoing and the PM task can also change the policy over time (Hicks et al., 2005; Marsh et al., 2005). Interestingly, Kuhlmann and Rummel (2014) recently showed that individuals can also flexibly update their attention-allocation policy after initial intention encoding as a result of learning during the ongoing task which trials are PM-relevant. Thus, these authors showed that participants who knew that PM cues would only occur in a sub-set of trials were able to learn which trials were PM-relevant as demonstrated by a reduction in ongoing cost for PM-irrelevant trials (see also Lourenço et al., 2013).

In addition, we presented participants with an atypical/typical category exemplar at both instructions and practice. Previous research suggests that learners’ metacognitive beliefs about how item characteristics affect memorability are sensitive to task experience (e.g., Tullis and Benjamin, 2012). By analogy, we assume that intention retrieval during practice may have strengthened participants’ beliefs about the difficulty/ease of successfully fulfilling the intention. Particularly in laboratory settings this might provide participants with additional information on which to base their expectations about PM demands since they lack prior experience with the PM task (cf. Rummel and Meiser, 2013). Future research could examine whether target exemplars presented at instructions (i.e., at the time of intention formation) guide attention allocation or whether metacognitive beliefs about the difficulty of completing the PM task are also determined by direct task experience during practice. Although the present study does not isolate the locus of the effect more precisely, it does demonstrate that information about particular target exemplars influences metacognitive expectations and thereby the amount of attention devoted to a categorical intention and task interference.

We argued that worse PM performance in the low relative to the high tacit demands condition was due to differences in attention allocation policies. Alternatively, it could be claimed that worse PM performance in the low demands condition (typical exemplars at encoding) was due to the mismatch between encoding and retrieval contexts (Tulving and Thomson, 1973). Participants might have generated animal exemplars at encoding (Ellis and Milne, 1996) and, because all PM targets were atypical animals, doing so would facilitate recognition of targets for individuals in the high demands condition only. Even if we assume that participants spontaneously generated category exemplars at encoding, and that in the high demands condition these were the same items later presented, the context matching account is inconsistent with our observation that tacit demands affected task interference prior to any target occurrence. If participants disregarded the information about the target exemplars when allocating attention to the PM task, there should have been no difference in the representation of the intention in memory and, accordingly, no cost differences between low and high tacit demands conditions. Therefore, the most parsimonious explanation of the results is that reduced PM performance for the low demands condition was primarily due to participants allocating insufficient resources to meet actual task demands.

Finally, note that by tacit demands we mean that we did not, at any point, directly instruct participants with respect to the demands of the PM task. Instead, demands were conveyed indirectly by providing participants with particular exemplars of PM targets before ongoing task performance. We are not claiming that the effect of target exemplars on attention allocation occurred without individuals’ conscious apprehension, although we acknowledge that this is a possibility. As proposed by Hicks et al. (2005, p. 442) “[t]he setting of an initial attentional allocation policy need not be conscious, but may represent a metacognitive strategy about how to approach the entire task set, and therefore, not necessarily be accessible to conscious awareness”.

In conclusion, three key aspects of the present study distinguish it from previous research. First, we manipulated tacit demands about the PM task. Second, objective PM task demands were the same for all participants. Third, for some participants tacit and actual PM demands were incongruent but participants were never explicitly warned about such change. Our findings suggest that in studying attention allocation policies and their impact on task interference in PM tasks it is important to consider the role of tacit information about PM task demands (e.g., as conveyed by specific target exemplars at encoding). Such information can influence individuals’ beliefs about the ease of fulfilling a PM task, as evidenced by its effect on ongoing task processing, and can harm PM performance when actual demands turn out higher than expected.

## Conflict of Interest Statement

The authors declare that the research was conducted in the absence of any commercial or financial relationships that could be construed as a potential conflict of interest.

## Footnotes

^1^Before being debriefed, participants in the PM groups were asked to fill in typicality ratings on a scale from 1 (very typical animal) to 5 (very atypical animal). As expected, *dog* and *mouse* (*M* = 1.5, *SD* = 0.4) were rated as more typical than *walrus* and *raccoon* (*M* = 3.9, *SD* = 0.6) and *puffin, gazelle, boar*, and *hyena* (*M* = 3.2, *SD* = 0.4), and the two PM groups did not differ in their ratings.

^2^Some readers might be concerned that this pattern could also be explained by monitoring being reinstated following detection of the first target (cf. Scullin et al., 2010, 2013). We therefore repeated this analysis while also including tacit PM demands (high, low) as a between-subjects factor. Although there was a reduced number of participants (six) missing the first target in the high demands condition, the three-way interaction was marginally significant, *F*_(1,54)_ = 2.83, *MSE* = 3,108.34, *p* = 0.098, *η_p_*^2^ = 0.05. In the high tacit demands condition (contrary to the low) there was no greater slowing from trials preceding to those succeeding the first target when target detection was successful (791 to 799 ms) relative to when it was unsuccessful (847 to 859 ms). These data are consistent with our interpretation of the results as reflecting an adjustment of the attention allocation policy following realization that targets could be atypical exemplars.
